# The use of the Socratic inquiry to facilitate critical thinking in nursing education

**DOI:** 10.4102/hsag.v24i0.1224

**Published:** 2019-04-23

**Authors:** Agnes Makhene

**Affiliations:** 1 Department of Nursing Sciences, University of Johannesburg, Johannesburg, South Africa

**Keywords:** dialogue, questioning, higher order thinking, critical thinking, Socratic

## Abstract

**Background:**

Critical thinking is a skill that nurse practitioners are required to have. Socratic inquiry can be used to facilitate critical thinking in nursing. Nurse educators seek methods to infuse into teaching content to facilitate students’ critical thinking skills, and one of such methods is the use of Socratic inquiry as a teaching method.

**Aim:**

This article aims to explore and describe how Socratic inquiry can be used to facilitate critical thinking in nursing education.

**Setting:**

This study took place in a nursing department at a university in Johannesburg.

**Methods:**

A qualitative, exploratory, descriptive and contextual design was used. Purposive sampling was used to draw a sample of 15 nurse educators determined by data saturation. Miles, Huberman and Saldaña’s methodology of qualitative data analysis was used. Lincoln and Guba’s strategies for trustworthiness and Dhai and McQuoid-Mason’s principles of ethical consideration were used.

**Results:**

Three main themes emerged: the context necessary for Socratic inquiry, dispositions in Socratic inquiry and strategies to use in Socratic inquiry to facilitate critical thinking skills of students.

**Conclusions:**

Socratic inquiry can be used both in education and practice settings to facilitate the use of critical thinking skills to solve problems.

## Introduction

Socratic inquiry refers to the kind of questioning in which the original question is responded to as though it was an answer (Zare & Mukundan 2015:256). It is a type of questioning that deeply probes or explores the meaning, justification or logical strength of a claim, and position or line of reasoning. This, in turn, forces the the one who first asked questions to reformulate a new question in light of the progress of the discourse. Questions that are asked investigate assumptions, viewpoints, consequences and evidence (Brookfield 2011:92–99). On the other hand, critical thinking is considered to be a necessary skill that nurse practitioners should have in order to function efficiently in an ever-changing healthcare setting. Patient care has become so diverse and complex to the extent that nurses are facing an ever-increasing demand to make decisions and solve problems which calls on the use of critical thinking. It has become even more important that students are taught to think critically. Critical thinking is a learnt skill, which means it can be facilitated during teaching and learning. The main focus has been on teaching critical thinking as a stand-alone subject and has since been essential to research on how to facilitate it as a skill. However, this topic is not explicitly taught or assessed within current programmes, yet the need is greater than ever, in an era of information explosion, spiralling healthcare costs, and increased understanding about metacognition to produce graduates who are critical thinkers (Huang, Newman & Schwartzstein 2014:95).

Therefore, the focus of this article is to describe how Socratic inquiry can be used to facilitate critical thinking in students. Facilitation refers to the promotion of critical thinking through the creation of an environment that is conducive to such thinking, using a dynamic interactive process (Theory for Health Promotion in Nursing, University of Johannesburg 2009), while critical thinking refers to a purposeful, self-regulatory judgement that results in interpretation, analysis, evaluation and inference, as well as explanation of the evidential, conceptual, methodological, criteriological or contextual considerations on which the judgement is based (Facione 1990:2). This article will assist nurse educators to use the Socratic inquiry in teaching and learning to facilitate critical thinking skills of students.

## Research design

This study applied a qualitative approach in the form of an exploratory descriptive design (Burns & Grove 2011:65) that was contextual in nature.

## Research methods

### Research setting

This study took place in a nursing department at a university in Johannesburg. The department offers 4-year undergraduate bachelor’s programme, post-basic 3-year bachelor’s degree, post-basic qualifications in nursing, for example, nursing education, community nursing science, advanced midwifery, among others, and master’s and doctoral degrees. The students include both undergraduate and postgraduate students. The department subscribes to the constructivist teaching and learning philosophy, whereby students are learning ‘to be’.

### Population sampling strategy

Participants were nurse educators who taught in the nursing department. The researcher approached the prospective participants on a face-to-face basis where the purpose of the study was explained and a request to participate was made. A non-probability purposive sampling method was used to draw the sample of 15 (*N* = 15) nurse educators from the lecturer population in the Faculty of Health Sciences. This method was appropriate as the researcher was looking for nurse educators who were willing to bring forth specific, rich and in-depth information related to the use of Socratic inquiry to facilitate critical thinking. The nurse educators were involved in teaching in the 4-year bachelor’s degree nursing programme and had a teaching experience of 5 years and above, which were the inclusion criteria for the sample. The sample size was determined by data saturation. No participants dropped out of the study. The participants gave informed written consent to participate and were made aware of their right to withdraw participation at any stage in the research without consequences.

### Data collection method

The researcher conducted 15 unstructured individual interviews at the convenience of each participant, as they were allowed to determine the date and time of interviews. The voice recording of the interviews was done with the consent of the participants. This enabled the researcher to use quotes from verbatim transcription of the interviews to enhance the credibility of the findings. Field notes were taken during and after the interviews to enrich the collected data. Follow-up questions were asked based on the responses of participants for clarification to enhance the depth of the data. Data collection was completed after data saturation was reached with the 15th participant. The interviews lasted for 30–60 min each as determined by the responses. The researcher posed the following central question to which the participant had to respond: ‘how can the Socratic inquiry be used to facilitate the thinking skills of students?’ If a response lacked sufficient detail, depth or clarity, the researcher followed up with a question, encouraging the participants to complete or clarify their response or asked for further examples and evidence. Based on the participant’s response, the researcher paraphrased, probed, clarified, reflected and summarised to gather comprehensive information and understanding of the participants’ responses.

### Data analysis

The total number of participants was 15 (14 women and 1 man). Their ages ranged from 35 to 62 years. There were five white, nine African and one mixed race participants. The sample included one professor, two senior lecturers and nine junior lecturers. This heterogeneous sample happened naturally as this was the staff establishment in the department. Miles, Huberman and Saldaña’s (2014:109–112) methodology of qualitative data analysis was used to analyse and draw themes from the data. Chunks of information were extracted from the transcripts, and the perceptions were grouped meaningfully and placed in matrices. Key information from the written chunks of information was identified so as to enable the researcher to easily get back to them in the content, should the need arise. Specific illustrations from written-up field notes were included. The researcher read through the transcripts to get original responses, while concentrating on similar patterns, feelings and thoughts. When data saturation was reached, similar patterns were grouped together to derive meaningful themes and categories. Content–analytical summary tables were used to clarify the researcher’s understanding. Conclusions were checked, confirmed and verified for accuracy. The data analysis protocol, audiotapes and field notes were given to an independent coder to analyse the collected data independent of the researcher to verify the accuracy of data analysis, which further increased the dependability and confirmability of the findings. The independent coder was purposively chosen as she had experience in qualitative design methodology, data analysis using matrices and held a doctoral qualification. A consensus meeting was held after she was done with the analysis. Five follow-up individual interviews were held with participants 4, 6, 8, 10 and 15 to establish the accuracy of the analysed data. This was done as a trustworthiness strategy to establish the dependability and credibility of the findings.

### Measures to ensure trustworthiness

Lincoln and Guba’s strategies for trustworthiness as described in Polit and Beck (2018:296–302), namely, credibility, dependability, transferability, confirmability and authenticity, were employed. Credibility was ensured through prolonged engagement where sufficient time was spent with each participant to gain in-depth understanding of how Socratic inquiry can be used to facilitate critical thinking. Member checking was done by taking some of the transcripts back to the participants to verify the accuracy of the collected data and to establish whether emerging interpretations were the accurate representation of what was said during data collection. This further ensured the credibility and dependability of the data. Follow-up interviews were conducted with some of the participants during data analysis to verify the accuracy of the collected data. The researcher ensured that data are consistent and neutral and the findings were subject to change and stability to attain dependability. Transferability of the findings in this study to other contexts was ensured by providing thick description of the context in which the study took place, the participants and research methods used. The value of the data was achieved through confirmability. Authenticity was ensured by voice recording of interviews and verbatim transcription of the data collected.

### Ethical considerations

The principles of autonomy, informed consent, beneficence, justice and non-maleficence were observed (Dhai & McQuoid-Mason 2011:14–15). Participants signed informed consent forms. The ethics committee of the University of Johannesburg gave ethical clearance for the study (Ref 35/05/04). Participants’ confidentiality and privacy were ensured in that their names were not mentioned in any of the data collected and nowhere in the description of the findings, and the interviews took place in each participant’s office.

## Findings and discussion

Data analysis led to the emergence of three main themes and subthemes relevant to each. These were a teaching/learning context for Socratic inquiry, attitude necessary during Socratic inquiry and strategies on how to use Socratic inquiry to facilitate critical thinking. The findings and field notes were integrated into relevant existing literature to enhance the richness of data and are discussed as such.

### The teaching/learning context for Socratic inquiry

The participants cited that it is important for the educator to create a teaching and learning environment that is conducive by ensuring that students feel psychologically safe, there is mutual respect and they are not intimidated by the questions asked.

‘It is essential that the educators ensure that the students feel “safe” to answer and ask questions without fear of being ridiculed or made to feel stupid.’ (Participant 2, 50 years old, female)

The nurse educator must be culturally aware and treat students with respect.

‘There must be mutual respect between students and the educator and sensitivity to cultural diversity.’ (Participant 5, 40 years old, female)

It emerged from the interviews that the teaching/learning context allows for full participation of each student without discrimination.

‘I think it is important that learning environment is such that there is mutual trust between the nurse educator and the students, and between the students themselves as this gives assurance to the students that they can freely participate in the teaching/learning process, and also trust that their viewpoints will be considered and taken seriously by others without bias or prejudice.’ (Participant 3, 46 years old, male)

The context that facilitates questioning should be one that allows for mutual respect. The educator needs to ensure that the teaching/learning environment is one where there is mutual respect among the students and between the students and the educator. The environment must allow for freedom of expression without prejudice or bias, and principles of democracy where everyone is treated equally should be established by the educator (Paul & Elder 2010:34–35). Students come from different cultural backgrounds; therefore, the teaching/learning environment must be one where the diversity is acknowledged, and there is cultural tolerance and accommodation. Brookfield (2011:92–96) is of the view that the learning environment should be one that encourages dialogue and the educator must allow for enough wait time during questioning. The educator should create an enabling environment and space for students to reflect when responding to inquiries. The learning environment must be less structured and emotionally supportive in order to prompt the learners to explore what they consider important. The appropriate behaviour of the nurse educator will enhance the perceived psychological safety of the learning environment. The nurse educator must avoid repeatedly interrupting students before they have finished responding; breaking eye contact with the responder; or using an aggressive or condescending tone of voice, facial expressions such as grimacing and/or alienating body language such as turning away from the responder will erode students’ sense of safety and self-worth. Students who do not feel safe or self-confident will not initiate responses to questions or will provide short or purposefully erroneous answers when called upon (Tofade, Elsner & Haines 2013:155).

### The attitude necessary during Socratic inquiry

According to the participants, there are certain attitudinal traits that both educators and students need to display when using the Socratic inquiry method in teaching.

‘The educator need to accept and accommodate each student’s capabilities as some students may feel the educator is out to get them when asking questions.’ (Participants 1, 47 years old, female)

‘The student must also not judge each other in their interaction in class as they engage with the questions asked and answers given and they must also be aware that they may be wrong in their responses and be prepared to adjust their thinking.’ (Participants 7, 53 years old, female)

‘It is important that the nurse educator displays a non-judgmental attitude. This will make the students to understand that they can make mistakes without being judged.’ (Participant 13, 60 years old, female)

Good Socratic inquiry takes in an atmosphere of mutual respect where there is expression of warm acceptance, understanding and non-judgmental attitude that will encourage students to engage in critical thinking. The student must be taught to value objectivity and rationality to resolve problems. They should respect evidence as the test for accuracy and display a willingness to suspend a judgement. They need to realise that their answers may be wrong hence the need to judgment without jumping at conclusions. Tolerance for ambiguity and an exhibition of a healthy scepticism, curiosity, and respect for the use of reason are further attitudinal attributes that are necessary in the engagement in Socratic inquiry. The student should be made aware that it is important avoid egocentric tendencies if their critical thinking skills are to be facilitated through Socratic inquiry (Brookfield 2011:92–96; Paul & Elder 2010:34–35). Questioning has both cognitive and affective components. Successful questioners value objectivity and rationality to resolve problems. They respect evidence as the test for accuracy, express willingness to suspend judgements and are tolerant of ambiguity to a point. These are the characteristics an educator should aim for in the facilitation of students’ critical thinking skills. Questioning is characterised by scepticism, curiosity and respect for the use of reasons (Rajput 2009:62–69). In this strategy, the educator has an obligation to guide the students as they formulate ways to gather information or evidence to answer questions. Depending on the variances in degree of assistance, students determine what data might be relevant, decide how to gather it, represent the collected data and organise it in a useful manner. Socratic inquiry is a method that will move the students towards the consideration of various options and the development of constructive alternatives through the use of their facilitated critical thinking skills in problem-solving.

### Strategies on how to use Socratic inquiry to facilitate critical thinking

The use of Socratic inquiry in the facilitation of critical thinking involves specific questions posed to illicit particular information. Questioning must compel thoughtfulness, evaluation and synthesis of facts and concepts. The participants said the nurse educator should ask questions in a manner that will make students think and question their own thinking patterns as well.

‘I normally ask questions with words such as “explain, compare, why, how did you get to that conclusion in order to get the students thinking. What is the best way to solve this problem and why, do you agree or disagree with this statement?” The questions should also be such that they force the students to evaluate assumptions, viewpoints, consequences and evidence.’ (Participant 11, 55 years old, female)

‘I use the Socratic method of questioning which focuses on clarification of what is said. Socratic questioning fosters critical thinking, evaluation, and knowledge application by the students. I find that this method of questioning probes beneath the surface of things and pinpoints problematic areas of their thinking processes. It encourages the student to become their own questioner and to develop habits of critical reflection.’ (Participant 9, 58 years old, female)

‘I also use a lot of thoughtful questioning in my teaching because through questioning I take the students from the known to the unknown as well as stimulate debate and argument which are facilitative of critical thinking. It is important that the questions that we ask are such that they stimulate higher order thinking, for example, evaluation and synthesis. For example, I ask questions like, “what is the problem here, how did you arrive at the solution, why the choice of solution, how can you do it differently next time?”’ (Participant 4, 50 years old, female)

The Socratic inquiry encourages learners to reflect and think independently and critically. It is practised in small groups with the help of a facilitator so that self-confidence in one’s own thinking is enhanced and the search for truth in answer to a particular question is undertaken in a common manner. The method begins by calling on a student at random and asking them about a central argument put forth by one of the other students. The questions can take several forms. Questioning must compel thoughtfulness, evaluation and synthesis of facts and concepts. According to Tofade et al. (2013:155), the educator should use the following guidelines when using Socratic questioning: must develop categories of questions such as exploratory, spontaneous, and focused questions. Exploratory questioning is used to find out how much students know about the issue under discussion. This type of question needs to be planned in advance and is used to introduce a new topic to students, review past discussions of a topic or determine how much students have retained from the previous learning sessions. Spontaneous questioning is best used when students are naturally curious about the topic or when an ongoing discussion slows as well as probing students’ thoughts in an effort to get them to explore their beliefs and assumptions. This type of question prompts students to self-correct, rather than be corrected by an educator, through the reflective process being used to analyse the question being asked. Spontaneous questioning can also be used when an important issue is raised, when students are on the edge of a breakthrough in learning or when discussion requires clarification. Focused questioning aims to bring attention to specific issues on which an educator would like the students to reflect while stimulating the students intellectually by forcing them to critically analyse and evaluate their thoughts and perspectives. It encourages the students to use the metacognitive process to analyse their own thinking processes (Kost, Frederick & Chen 2015:23–24; Zare & Mukundan 2015:260).

An educator should begin the inquiry process by posing an open-ended question to students. The students are encouraged to adhere to a subsidiary question until it is answered while avoiding coercion and manipulation. The students must be gently nudge and guided to examine the issues they take for granted, such as assumptions, beliefs, experiences and paradigms. Respond to all answers with a further question in order to develop their fuller thinking and depth of thinking (Billings & Halstead 2012:274–275). Treat all students’ assertions as in need of development and connecting points to further thoughts and recognise that any thought can exist fully in a network of connected thoughts. Students can be given pre-class assignments that will lead to adequate preparation for class. Ask ‘why’ questions that require explanation of principles and help to determine the amount, direction and quality of the student’s thinking (DeWaelsche 2015:140–147). Paul and Elder (2010:34–35) are of the opinion that an educator should assist students to form relationships, induce involvement and enhance the learner’s critical thinking through questioning. The questions should be designed to assess various cognitive skills and sub-skills associated with critical thinking. The students’ verbal and non-verbal responses as well as the flow of questioning should be monitored. Stimulate mental alertness and encourage co-operative questioning through questions generated by the students. The educator should pose questions to create an awareness of a point of view in the students’ minds that they may have overlooked, to further create doubt; the objective is that they test their proposition anew. Ensure that the students are clear about what is being said by testing it against their individual experiences and asking clarity seeking questions to establish a reference to experience and to avoid judgment of too general a nature (Hughes & Quinn 2013:165). Paul and Elder (2010:34–35) argue that the educator should encourage intellectual perseverance in the face of difficulty but, on the other hand, display intellectual humility to accept temporarily that their thinking and dialogue may take a different course. Guide them to adopt justified positions and formulation of own thoughts as an answer to a question. Restrain oneself from providing answers by allowing the students to discover insights on their own and to independently seek information and formulate criteria to clarify issues or arguments for assessment and making judgements. The educator should raise questions that are investigative in nature and of a fundamental nature: questions about the significance of basic elements of a subject; and questions seeking explanations of basic patterns -– what is causality? Encourage the students to express their thoughts clearly to be understood by others, and to grasp the thoughts of others. Insist on precise and shared understanding. The act of directing the thinking of the students should never encroach on the student’s emerging judgement (Tallent & Barnes 2015:435–441). Torabizadeh, Homayuni and Moattari (2018:174–185) assert that the core of Socratic inquiry is that typically there is more than one ‘correct’ answer, and more often, no clear answer at all. The primary goal of Socratic inquiry in the learning area is not to answer usually unanswerable questions, but to encourage the students whose critical thinking is facilitated to explore different aspects of answers brought forth and their justification. This method encourages the student to move beyond memorising the facts and instead focus on the application of developed critical thinking skills in solving problems at hand. According to Knezic et al. (2010:1104–1111), the Socratic inquiry means that a student is involved in a dialogue, starting with the concrete and remaining in contact with concrete experience. Insights will be gained only when, in all phases of a Socratic dialogue, the link between any statement made and personal experience is explicit. This means that a Socratic dialogue is a process that concerns the whole person. The students should be encouraged to focus on a subsidiary question until it is answered. To achieve this, the students are required to bring great commitment to their work, and to gain self-confidence in the power of reason. This means, on the one hand, not giving up when the task is difficult but, on the other, to be intellectually humble enough to accept, for a time, a different course in the dialogue to return to the subsidiary question. Striving for consensus will require an honest examination of the thoughts of others and the student being honest in their own statements (Torabizaden et al. 2018:174–185). There are certain types of Socratic questions that should be asked to gather specific responses or information. Socratic questioning or inquiry encourages to question more systematically and deeply. Examples of Socratic questions as adapted from Paul and Elder (2008:np) are described.

Question to seek clarity:

- What do you mean?
- How does cyanosis relate to difficulty in breathing?

Questions to probe assumptions

- You seem to be assuming that a patient who is presenting with dyspnoea is having a respiratory condition….do I understand you correctly?
- What is your assumption with regard to decreased oxygen saturation and inflammation of the respiratory tract?

Questions probing reasoning and evidence

- How do you know that decreased SATS result from inadequate respiration?
- Do you have evidence to support your reasoning?

The study found that it is important to create a conducive environment if one is to use Socratic inquiry to facilitate critical thinking. It was further found that both the nurse educator and student nurses must possess specific attitudinal traits and that there are strategies that need to be employed in order to facilitate critical thinking through Socratic inquiry.

## Conclusion and recommendations

The aim of this study was attained. Socratic inquiry can be used as a teaching and learning strategy to facilitate the critical thinking skills of students. The findings were that there is a specific teaching–learning environment that the nurse educator must create; the educator and students need to demonstrate certain attitudinal dispositions in order to be successful in using Socratic inquiry as a teaching strategy to facilitate critical thinking of students. A teaching–learning environment characterised by an open, mutual respect and trusting relationship should be ensured. Therefore, the implication is that the educator needs to take adequate time to construct thought-provoking questions and aim at facilitating a discussion that follows a good questioning exercise. Furthermore, the facilitation of students’ critical thinking skills will be enhanced if a pre-class assignment that leads to adequate student preparation is designed, and further research can be conducted to test if pre-class assignments prepare students for the facilitation of their critical thinking skills. The educator can use questioning spontaneously as an exploratory strategy, or with issue-specific content. The educator’s role is mainly of asking questions and providing support for the students facilitated critical thinking skills. Student-initiated questions are also encouraged as they increase higher order thinking which requires them to analyse information, clarify meaning and draw inferences by examining relations between concepts and justifying their responses which are attributes of critical thinking. Socratic inquiry, if properly used, can show that decisions are usually conscientiously made, and emanate from particular premises, beliefs and conclusions that are the subject of justified argumentation. Students will learn to discover the structure of their thoughts and to develop sensitivity to clarity, accuracy and relevance. It also assists them to arrive at judgements based on their own reasoning, and to note claims, evidence, conclusions, interpretations, implications, concepts and points of view that are considered to be elements of critical thinking. It is further recommended that this teaching methodology should be used both in the classroom and clinical context to facilitate students’ critical thinking skills.
